# Physiological Explanation of the Beauty of Form

**Published:** 1839-01-01

**Authors:** 


Physiological Explanation of the Beauty of Form. By *Benjamin F. Joslyn*, M.D, Professor of Natural Philosophy and Lecturer on Anatomy and Physiology in Union College, N. Y. 8vo. stitch'd, pp. 29.

This Lecture is a very ingenious attempt to explain beauty of form physiologically.

The explanation is based, as our author informs us, on the proposition, that the action of every muscle is attended with a sensation which is at first agreeable, but which, if the action is continued for a short time with intensity and without intermission, becomes painful. That there is pleasure attending those varied motions which depend upon the actions of different muscles in succession after intervals of rest in each, we know from our own consciousness as well as from that instinctive propensity to play which we observe in children and young animals. That the prolonged action of a muscle is painful, we may readily convince ourselves by endeavouring to hold the arm for some time at right angles with the erect trunk. With the arm in this position, a pound weight on the hand or even the weight of the arm itself becomes in a few minutes almost insupportable. We presently begin to feel pain in the shoulder and anterior part of the arm, the former from fatigue of those muscles which originate from the scapula and keep the os humeri elevated, and the latter from fatigue of the muscles which originate from the scapula and os humeri, whose muscular fibres are in front of the os humeri, and by their contraction elevate the fore arm in consequence of their tendinous attachment to its bones. Yet a man may labor all day with his arms without this painful sensation; because a muscle requires but momentary rest, in order to regain that degree of energy which is momentarily lost by action.

Thus a muscle may be considered as having a simple action, which cannot be sustained with uniformity a minute of time without actual pain, nor a second of time with positive pleasure. Of course this is speaking off book. But to express the law in more general terms, as we diminish the duration of a muscle's action we diminish the pain until we arrive at an action whose attendant sensation is neutral, *i. e.* neither painful nor pleasurable ; as soon as we have passed that point and have begun to execute motions a little more transient, the attendant sensation becomes positively pleasurable, and the pleasure increases as the separate actions become more transient.

Of the general correctness of this principle we think that there cannot be any doubt. We proceed to its application to the solution of Beauty of Form.

It needs no argument to shew that our ideas of form are obtained from the operation of two senses—touch and sight. Part of the sensations which we refer to each result from the muscular sense, that is from the sensation of action in those muscles employed in the apparatus of each.

Observe, for example, the determination of the form of a solid by the eye. The solid is bounded by lines.

For examining the form of a line, as well as that of the outlines of any other object, it is necessary to direct the optic axis to its different points in succession; this is always done in a first and critical inspection, and in many instances in which we are unconscious of the motion. Hence, as the actions of the four muscles situated respectively at the lower, outer, upper and inner parts of the eye, effect all its voluntary motions, it is essential in determining by the eye, the form of a line, that one or more of these muscles shall direct the axis successively to its different points.

In the ordinary state of the muscular system, and within certain limits, the motion of the eye in any direction is pleasurable, as compared with that inaction of the muscles attending indolent vision in a fixed direction, or with that incessant and equal, but not necessarily energetic action of them all, requisite for the preservation of their equilibrium during the accurate and prolonged inspection of a minute dot whose figure is inappreciable. Thus the tracing of a line will, to a certain extent and for a certain time, afford some degree of positive pleasure; in other words, any short line will possess some degree of positive beauty. But a point awakens no such emotion, and can possess no beauty.

"When the head is erect, in examining a *straight horizontal* line we employ one of the lateral recti; if the line be vertical we employ the rectus inferior or superior. In either case, but one muscle acts, and that continuously. The muscle is not relieved, and its action is not attended with the maximum amount of pleasurable sensation. When the vision has been extended along the whole line, if we then immediately proceed to examine it in the opposite direction, the opposite rectus must at once exert a force sufficient to overcome the *momentum* of the eye-ball, and then exert a *continuous* action. Both these circumstances are unfavorable to pleasure. If the line is *oblique*, one lateral together with one inferior or one superior muscle is exerted, and the same principles which have been applied to the single muscles, apply to the muscles acting in pairs; the muscles of each pair acting simultaneously and continuously, and being, in the case of repeated examinations, opposed by the momentum of the eye, or in other words, the *inertia* of the eye-ball in motion. Oblique lines should be rather more agreeable than vertical or horizontal ones, as two muscles act together. The fact of the superior beauty of lines inclined to the vertical, so far from being explained, has not, perhaps, been stated. From the physiological theory, we should not expect to observe the same difference between them as between any straight line and a regular curve; for the occasional repose of a muscle conduces more to its relief than the simultaneous and uniform co-operation of *another* muscle; and the advantage which an oblique line has over a horizontal one in regard to intrinsic beauty, is in numerous instances compensated by extrinsic relations." 14.

It may readily be imagined how, by this hypothesis, curved lines are beautiful. In viewing a regular curve, no muscle of the eye-ball acts continuously and uniformly, but enjoys partial relief by remissions, or total relief by intermissions of its action; and the regularity of these remissions and intermissions, as well as the equal distribution of exercise, is promoted by the regularity of the curve. Acting in succession, the muscles afford mutual relief after actions of such short duration and variable intensity, as to afford positive pleasure; and in this *muscular pleasure* of the eye consists the *beauty of configuration.*

"The successive and accurate survey of distant points is not however invariably requisite to a degree of similar pleasure, in viewing a figure of such small angular extent as to be instantly recognized by one location of its image, as analogous to a larger one whose survey has directly afforded muscular pleasure.

Although I thus recognize the influence of association, the facts of this very case afford an interesting confirmation of the physiological theory; for a large circle or ellipse is more beautiful than one of diminutive size. The beauty of the one is original, its influence is direct; the beauty of the other is in part borrowed, and this part is weakened by reflection. Or to express it more literally, the one excites a pleasurable sensation, the other suggests a similar idea; the one affords a *perception,* the other a *conception* of beauty. Such, even with similar color and brilliancy, would be the difference between the full moon and a circular dot (·) or period; such the difference between a rainbow and a diminutive arc (⌒), a short accent inverted. Here the critic might be inclined to charge us with confounding the beautiful with the sublime. But the fact is, that criticism has constructed the sublime—as it has the beautiful—from heterogeneous materials, one of which is identical with one of the elements of beauty, and should in a physiological arrangement, be referred to the same class. In many instances a magnifying instrument will disclose minute irregularities and blemishes; but in every other case, physiology would show, that within certain limits, to magnify a beautiful *object* is to *magnify beauty.*" 16.

We need not enter on the analysis of the beauty of different curved lines. But before we close this notice, we may direct attention to Dr. Joslyn's account of the "Principle of Symmetry." He considers it under the heads of 1st, geometrical symmetry, or symmetry of form; 2nd, of symmetry of position.

*Symmetry of form,* he says, though implied in geometrical regularity is not identical with it, and requires a separate consideration. The beauty of forms geometrically symmetrical, in contradistinction from those deficient in the correspondence of opposite halves, depends upon two similar series of actions in different pairs of muscles. For example, the survey of an ovate leaf, or indeed that of almost any vegetable leaf,—so numerous are the provisions for our gratification—requires for its opposite halves two series of muscular actions, the different parts of the one corresponding with those of the other in duration, intensity and order of succession. The gratification in this case results from the harmony of muscular sensations individually pleasurable. The agreeableness of this harmony may depend upon a principle more psychological than that of the agreeableness of its elementary sensations. Yet the former is to a certain extent susceptible of a physiological generalization.

Dr. Joslyn offers some further observations upon this head, but we do not consider it necessary to follow him. Nor can we accompany him in his examination of the *symmetry of position.* His remarks on the final cause of this power in the human eye to appreciate beauty of form are sensible, and, being brief, may be quoted.

"The benevolence of the Author of Nature is strikingly manifested in connecting present pleasure with obedience to the natural laws. It has been shown that vision is attended with muscular action which is generally pleasurable. If seeing had required no muscular action, we should have wanted one of our present stimuli to the acquisition of knowledge. This stimulus is especially necessary in infancy, and then powerfully prompts to observation, even anterior to the dawnings of intellectual curiosity, with which it subsequently co-operates. We see, in this arrangement, the exemplification of a principle which extensively pervades the laws under which we are placed by the Creator—which is, that mental attainments, as well as other acquisitions, shall require action; and that action shall be attended with pleasure. Whether the acquisition is to be made by the manual labor of the artizan, by the manipulations of the artist, the chemist or the experimental philosopher, by the sedentary student of books, or by the observer of natural phenomena in his original survey of the universe, in every case it is muscular action." 26.

And, after pointing out the regular, curved, and symmetrical objects, and motions in which Nature delights, he adds:—

"All this beauty had been lost to man, but for a property of the eye, which, on a superficial reflection, might seem a defect. It is no less true than paradoxical, that the perception of these beauties depends on *indistinctness of vision*. To a being so constituted as to see with equal distinctness by oblique and direct vision, the same forms might be presented, but not as forms of beauty. Has the Creator, then, sacrificed a portion of our perceptive powers to our sensual gratification? I answer no. Has he, then, sacrificed a portion of our *direct* means of acquiring knowledge, to afford an incitement which should ultimately and indirectly enhance our attainments? Again I am compelled to answer in the negative. There is, in this arrangement, no intellectual sacrifice whatever, direct or indirect. This indistinctness of oblique vision, which might seem a defect, I consider an excellence. A simultaneous and distinct impression received from the whole field of vision, would distract the attention and preclude a minute and accurate examination of any particular part. But as our eyes are so constituted as to receive a strong and distinct impression only from the images of those objects toward which their axes are directed, and as our minds are so constituted that we can in a great measure neglect the weaker or less distinct impressions, we are able to acquire a more exact knowledge of any part of the field to which we choose to attend. To see every thing at once, would be to examine nothing. Such a constitution of the eye would be to vision what an indiscriminating memory is to the understanding." 28.

There is certainly great ingenuity, and probably much truth in the preceding views. We will not say that the explanation of Dr. Joslyn meets the whole case, nor are we convinced that the mind deserves to be excluded, so much as it is, from the determination of beauty of form. But we have selected the more prominent portions of Dr. Joslyn's argument, and laid them before our readers undiluted with comments of our own. Dr. Joslyn is evidently a very observant and a very intelligent man.

